# Unclassified fluent variants of primary progressive aphasia: distinction from semantic and logopenic variants

**DOI:** 10.1093/braincomms/fcac015

**Published:** 2022-02-02

**Authors:** Hiroyuki Watanabe, Sakura Hikida, Manabu Ikeda, Etsuro Mori

**Affiliations:** Department of Behavioral Neurology and Neuropsychiatry, Osaka University United Graduate School of Child Development, Suita, Japan; Department of Psychiatry, Osaka University Graduate School of Medicine, Suita, Japan; Brain Function Center, Nippon Life Hospital, Osaka, Japan; Department of Psychiatry, Osaka University Graduate School of Medicine, Suita, Japan; Brain Function Center, Nippon Life Hospital, Osaka, Japan; Department of Psychiatry, Osaka University Graduate School of Medicine, Suita, Japan; Brain Function Center, Nippon Life Hospital, Osaka, Japan; Department of Behavioral Neurology and Neuropsychiatry, Osaka University United Graduate School of Child Development, Suita, Japan; Brain Function Center, Nippon Life Hospital, Osaka, Japan

**Keywords:** Alzheimer’s disease, anomic aphasia, logopenic progressive aphasia, transcortical sensory aphasia, Wernicke’s aphasia

## Abstract

Primary progressive aphasia, a neurodegenerative syndrome, presents mainly with language impairment. Both semantic and logopenic variants are fluent variants of primary progressive aphasia. Before the research criteria of primary progressive aphasia were proposed, progressive fluent aphasias, such as progressive anomic aphasia, transcortical sensory aphasia and Wernicke’s aphasia, were reported as classical progressive fluent aphasias seen in Alzheimer’s disease. However, since the research criteria of primary progressive aphasia were established, classical fluent variants (other than semantic and logopenic variants) have been neglected and have not been included in the current classification of primary progressive aphasia. This study aimed to determine whether unclassified fluent variants (other than semantic and logopenic variants) can be manifestations of primary progressive aphasia. This study also reconfirmed the characteristics of classical progressive fluent aphasia, such as progressive anomic aphasia, progressive transcortical sensory aphasia and progressive Wernicke’s aphasia as unclassified fluent variants of primary progressive aphasia, using comparison with the current model of primary progressive aphasia. Twelve consecutive patients with an unclassified fluent variant other than semantic or logopenic variant underwent language, neurological, neuropsychological and neuroimaging (MRI and single-photon emission computed tomography) testing. Based on comprehensive language tests, we redefined the diagnoses as primary progressive anomic aphasia (*n* = 8), primary progressive transcortical sensory aphasia (*n* = 3) and primary progressive Wernicke’s aphasia (*n* = 1). Anomic aphasia was characterized by anomia but preserved repetition and comprehension; transcortical sensory aphasia by relatively preserved repetition but poor word comprehension; and Wernicke’s aphasia by poor repetition and word comprehension. In patients with anomic aphasia, voxel-based morphometry of MRI data revealed cortical atrophy, which was most prominent in the temporoparietal lobes, with no obvious lateralization; in two-thirds of patients with transcortical sensory aphasia and in one patient with Wernicke’s aphasia, it revealed atrophy, predominantly in the left temporoparietal lobe. Statistical analysis of single-photon emission computed tomography using three-dimensional stereotactic surface projections revealed patterns of left-sided hypoperfusion in the majority of patients. The temporal and parietal lobes were involved in all cases; the degree of hypoperfusion was higher in patients with transcortical sensory aphasia or Wernicke’s aphasia than in patients with anomic aphasia. The present study demonstrated the clinical and imaging features of 12 patients with an unclassified fluent variant of primary progressive aphasia, which we redefined as primary progressive anomic aphasia, primary progressive transcortical sensory aphasia and primary progressive Wernicke’s aphasia. Classical fluent variants other than semantic and logopenic variants can be found in primary progressive aphasia.

## Teaser

Watanabe *et al*. reconsidered the existence of unclassified fluent variants of primary progressive aphasia (namely primary progressive anomic aphasia, primary progressive transcortical sensory aphasia and primary progressive Wernicke’s aphasia) that are distinct from semantic and logopenic variants, and provided insights into the spectrum of primary progressive aphasia.

## Graphical Abstract

**Figure:**
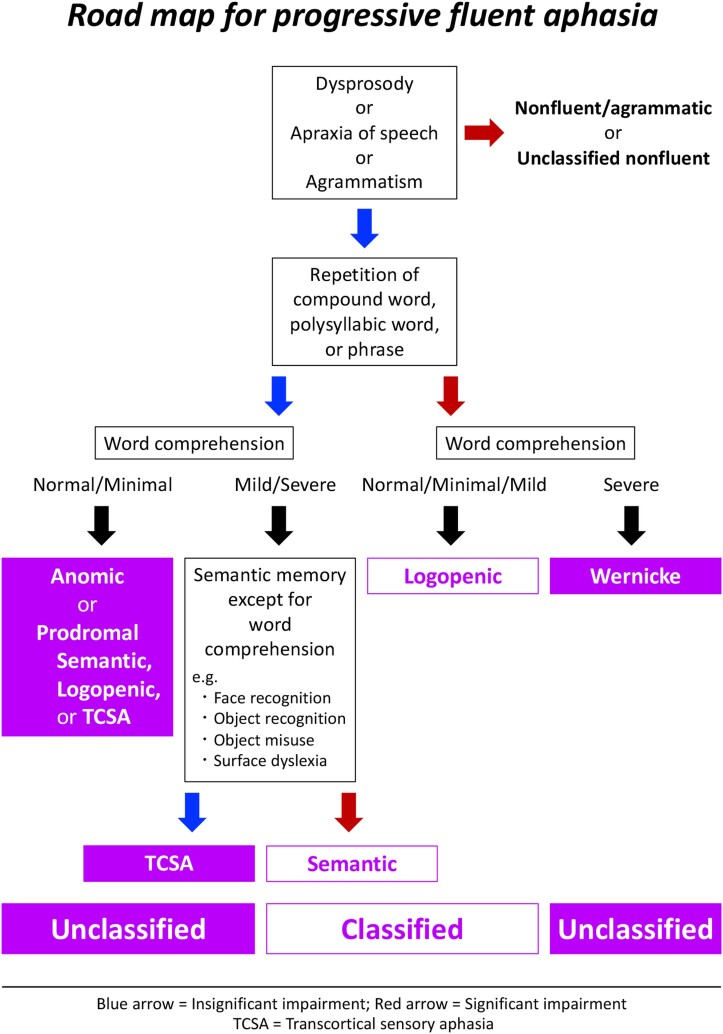

## Introduction

Primary progressive aphasia (PPA), which was defined by Mesulam,^1^ is a neurodegenerative syndrome, characterized by the early and relatively predominance of language impairment. Historically, since progressive and isolated language impairment in neurodegenerative disease was first described by Pick in 1892, schemes have been developed for the subcategorization of frontotemporal dementia (FTD).^2^ In 1998, the clinical diagnostic criteria for FTD were proposed,^3^ when the FTD spectrum was divided into three clinical variants including two current forms of PPA: the behavioural variant, non-fluent aphasia variant and semantic dementia variant.^2^ In 2004, ‘logopenic progressive aphasia’ as a third subtype of PPA was proposed by Gorno-Tempini *et al*.^4^ In 2011, an international group proposed the research criteria for categorization of PPA into three clinical variants: non-fluent/agrammatic variant of primary progressive aphasia (nfvPPA), semantic variant of primary progressive aphasia (svPPA) and logopenic variant of primary progressive aphasia (lvPPA).^5^ PPA is associated with multiple neuropathologic entities such as all major forms of frontotemporal lobar degeneration as well as Alzheimer’s disease.^6^ Especially, lvPPA is widely known as progressive fluent aphasia associated with Alzheimer’s disease.^5,7^ Here, we use the term ‘fluent’ to characterize the following speech: apraxia of speech or agrammatism is absent; and prosody, grammar, syntax and phrase length are normal on spontaneous speech. The term ‘PPA’ is used for characterizing progressive and initially isolated aphasia in all neurodegenerative diseases.

svPPA and lvPPA are accepted as the fluent variants of PPA.^5^ Before the research criteria of PPA^5^ were proposed, progressive fluent aphasias, such as progressive anomic aphasia, progressive transcortical sensory aphasia (TCSA), and progressive Wernicke’s aphasia, were reported more frequently as those observed in Alzheimer’s disease than progressive conduction aphasia, which corresponds to current lvPPA with an impaired repetition.^8–11^ Patients with Alzheimer’s disease present most frequently with progressive anomic aphasia in the early stage of the disease, whereas progressive TCSA and Wernicke’s aphasia are seen most frequently in the late stage. However, since the research criteria of PPA^5^ were established, fluent variants of PPA (other than svPPA and lvPPA) have been neglected and have not been included in the current classification of PPA.

Several studies have suggested that some fluent variants of PPA are not classifiable as lvPPA or svPPA,^12–24^ indicating that the established research criteria^5^ may not account for the full range of clinical variants of PPA. This study aimed to determine whether unclassified fluent variants other than svPPA and lvPPA can be manifestations of PPA. We also reconfirmed characteristics of classical progressive fluent aphasias, such as anomic aphasia, TCSA and Wernicke’s aphasia as unclassified fluent variants of PPA, through comparison with the current model of PPA. In addition, we compared the neurological, neuropsychological and neuroimaging features of patients with these conditions.

## Materials and methods

### Subjects

We enrolled consecutive patients diagnosed with an unclassified fluent variant of PPA other than svPPA or lvPPA at the Nippon Life Hospital and Osaka University Hospital, Japan, between September 2018 and March 2020. The inclusion criterion was the diagnosis of PPA according to the PPA criteria.^5^ The exclusion criteria were as follows: (i) patients diagnosed with nfvPPA, svPPA or lvPPA; (ii) patients with a significant impairment of cognitive domains other than language; (iii) a history of other neurological diseases, psychiatric diseases or hearing impairment and (iv) any evidence of focal brain lesions other than atrophy on MRI. All patients were evaluated by an experienced behavioural neurologist or neuropsychiatrist, underwent standard neuropsychological and speech and language assessment by an experienced speech and language therapist and clinical neuropsychologist, and underwent routine laboratory investigations. MRI and *N*-isopropyl-*p*-[^123^I] iodoamphetamine single-photon emission computed tomography (SPECT) were also performed. The diagnostic classification was blinded to the results of the imaging analysis and was based on the results and recordings of the speech and language assessments, as well as samples of general conversation, which were reviewed in a consensus meeting 2–4 weeks after the clinical assessments.

All patients and their caregivers provided written informed consent. The study was conducted according to the principles of the Declaration of Helsinki. The study was approved by the ethics committees of the Nippon Life Hospital and Osaka University Hospital.

### Background neuropsychological and behavioural assessments

To determine the features of cognitive and behavioural alterations, all patients underwent the following tests: the Clinical Dementia Rating (CDR),^25^ the Mini-Mental State Examination (MMSE), the Addenbrooke’s Cognitive Examination-Revised (ACE-R),^26,27^ the Alzheimer’s Disease Assessment Scale (ADAS),^28,29^ the auditory pointing subtest on the famous-face recognition task of the Visual Perception Test for Agnosia (VPTA),^30^ and digit span and spatial span tests.

The following questionnaires were administered to the patients’ caregivers: the Neuropsychiatric Inventory (NPI) 12-item version^31^ for the assessment of behavioural and neuropsychiatric symptoms and the Cognitive Fluctuation Inventory^32^ to determine fluctuations in cognition.

### Language assessments

After bedside language assessments,^33^ the following in-depth evaluations were conducted: the Western Aphasia Battery (WAB),^34,35^ which served as the primary measure of global language ability; the Token test, used as a measure of long and syntactic comprehension ability and the Test of Lexical Processing in Aphasia (TLPA).^36^ The TLPA is a standardized, widely used language test for Japanese speakers, and a total of 200 items in line-drawing cards are included in the picture-naming task or the auditory word comprehension task. In the TLPA-word comprehension task, after listening to a spoken word, patients are asked to match one of the 10 line-drawing cards. The TLPA-word comprehension task was used to classify the severity of single-word comprehension impairment. TLPA-word comprehension scores were categorized as follows: normal, 198–200; minimal impairment, 181–197; mild impairment, 161–180 and severe impairment, <161.

### Determination of the extent of atrophy

The extent of atrophy on MRI was determined by the Brain Anatomical Analysis using Diffeomorphic Anatomical Registration through Exponentiated Lie Algebra (DARTEL) (BAAD) (http://www.shiga-med.ac.jp/∼hqbioph/BAAD(English)/BAAD.html).^37^ This software tool performs the voxel-based morphometry (VBM) analysis using the statistical parametric mapping (SPM). It has been developed for an automated program of the full process for the VBM analysis, and quantitatively calculates the extent of brain atrophy based on a comparison with MR images from the IXI database of age-matched healthy controls (Information Extraction from Images, Control Group IXI60 and IXI7080). The BAAD integrates several programs that work on the SPM software such as MarsBaR, Wfu_Pickatlas and XjView. Moreover, the BAAD uses maximum-likelihood estimation and maximum a-posteriori algorithms for accurate segmentation, and the MarsBaR re-calculates *t*-values within each region, to avoid any masking threshold effect to improve the accuracy.^38^ Regions of interest (ROIs) were set according to the Automated Anatomical Labeling, Brodmann areas and Laboratory of Neuro Imaging Probabilistic Brain Atlases based on the Montreal Neurological Institute (MNI) coordinate system. Total intracranial volume, age and sex were included as confounding covariates. The BAAD converts *t*-values to *Z*-scores and displays *Z*-scores of MNI anatomical ROI, in the form of both numerical data and false-colour graphics. *Z*-scores for regional volume were calculated using the following equation: *Z*-score =  [(control mean) – (individual value)]/(control standard deviation). We used a *Z*-score of 2 as the cut-off value in each voxel and voxels with a *Z*-score of ≤2 were considered voxels without significantly decreased regional volume.

### Determination of the extent of hypoperfusion

The extent of hypoperfusion on the SPECT was determined by 3D stereotactic surface projections (SSPs),^39^ where SPECT images were sampled at 16 000 predefined cortical locations and projected on the 3D image after realignment, spatial normalization and non-linear warping. The voxel values of an individual’s SPECT data were normalized to the whole-brain tracer uptake and compared with an age-matched normal database, yielding a 3D SSP *Z*-score image; the abnormalities of cerebral hypoperfusion were displayed with a *Z*-score map. *Z*-scores were calculated using the following equation: *Z*-score = [(control mean) – (individual value)]/(control standard deviation). We used a *Z*-score of 2 as the cut-off value in each voxel and voxels with a *Z*-score ≤2 were considered voxels without significantly decreased regional cerebral blood flow.

### Data availability

The raw data supporting the conclusions of this article will be made available upon reasonable request from the corresponding author.

## Results

### Demographic data and performances on neuropsychological and behavioural assessments

Twelve patients from the Brain Function Center of the Nippon Life Hospital (*n* = 9) and the Department of Psychiatry of the Osaka University Hospital (*n* = 3) met the inclusion criteria for the study (Table 1). During the same time period, six patients were excluded due to a diagnosis of nfvPPA (*n* = 4), svPPA (*n* = 1) and lvPPA (*n* = 1). For clarity, case numbers were rearranged in the order of the picture-naming task performance. Nine patients were women, and all but one subject (Case 6) were right-handed. The mean age of onset was 75.0 ± 7.3 years [mean ± standard deviation (SD)] with the onset of symptoms before 65 years of age occurring in one patient only. The majority (7/12) had experienced language impairment for a duration of ≤3 years, while the remaining five patients had experienced language impairment for a duration of ≤5 years. All patients visited us because of gradually progressive difficulty in speaking, which, in some patients, was followed by difficulty in auditory comprehension. The patients retained awareness of their language impairments, and their motivation to communicate was well preserved. None of the patients had a family history of any neurological disease.

**Table 1:** Demographic information and neuropsychological test scores

Characteristics	Case no.											
	1	2	3	4	5	6	7	8	9	10	11	12
CDR total score (0–3)	0.5	0.5	0.5	0.5	0.5	0.5	0.5	0.5	0.5	0.5	0.5	0.5
Sex	W	W	W	M	W	M	W	W	W	W	W	M
Handedness	R	R	R	R	R	L	R	R	R	R	R	R
Education	12	16	12	9	12	12	12	12	14	14	12	12
Duration from onset (years)	2	5	2	5	2	4	2	2	5	3	4	3
Age at onset (years)	82	69	75	74	68	77	83	82	83	68	61	71
MMSE total score (30)	28	28	28	**24**	**23**	**19**	**22**	**16**	**16**	**19**	**14**	**12**
ACE-R total score (100)	87	78	**73**	**63**	**61**	**62**	**65**	**47**	**63**	**49**	**44**	**38**
Digit span	F5B4	F5B4	F5B3	F5B3	F6B4	F6B3	F5B2	F5B3	F5B5	F5B3	F5B3	**F3B2**
Spatial span	F5B5	F7B6	F5B5	F5B5	F5B4	**F3B3**	F5B5	**F4B4**	F4B6	F5B5	F5B5	F5B4

The maximum score is noted in each row header. Boldfacing represents values that are considered abnormal. ACE-R, Addenbrooke’s Cognitive Examination-Revised; B, backward; CDR, Clinical Dementia Rating; F, forward; M, man; MMSE, Mini-Mental State Examination; W, woman.

The results of physical and neurological examinations and routine laboratory tests were unremarkable. Overall, MRI revealed cortical atrophy in the temporoparietal lobe. The medial temporal lobe was more or less atrophic in all patients. In addition, atrophy in the posterior cingulate cortex was apparent in four patients (Cases 1, 4, 5 and 12), and atrophy in the precuneus was apparent in six patients (Cases 2–6 and 12). SPECT revealed various degrees of left hemisphere dominant hypoperfusion in right-handed patients, and this observation is discussed in detail below. In addition, hypoperfusion in the bilateral left posterior cingulate cortex and precuneus was apparent in nine patients (Cases 1, 2, 4–6 and 9–12). Amyloid PET was performed in one patient (Case 11), in whom the [^18^F] flutemetamol PET imaging result was positive.

None of the patients showed significant generalized dementia (CDR score: 1–3) as measured by the CDR (Table 1). Episodic memory for daily events was well preserved in all patients. Regarding semantic memory, identification of famous or familiar people, visual object recognition, environmental sound recognition and object use were well preserved in all patients. Furthermore, no caregivers described the loss of object recognition beyond the naming impairment of the patients in daily activities, such as difficulty finding an object in their sight or not knowing how to use it. None of the patients had a score of >2 SD below the mean on the word recognition subtest of the ADAS, the visuospatial subtest of the ACE-R, the reading five irregular words subtest of the ACE-R and the famous-face recognition subtest of the VPTA (Supplementary Table 1).^27,30,40^ These findings indicated that memory function, visuoperceptual and visuospatial function, and non-verbal conceptual knowledge were relatively well preserved in all patients. Only 3 of the 12 patients showed mild behavioural or psychiatric symptoms as measured by the NPI (Supplementary Table 1).

### Features of aphasias

Language features are summarized in Tables 2 and 3. The mean ± SD of the WAB-Aphasia quotient score was 81.3 ± 9.2. Spontaneous speech was fluent in all patients; there was no evidence of dysprosody, apraxia of speech or agrammatism, and the WAB-Fluency score was ≥8. In addition, none of the patients showed buccofacial apraxia or limb apraxia. Therefore, none of the patients had a diagnosis consistent with nfvPPA (Table 3).^5^

**Table 2:** Performance in language tests

Characteristics	Case no.												Normative data, mean (SD)
	1	2	3	4	5	6	7	8	9	10	11	12	
*WAB*													
Aphasia quotient (100)	91.8	88.8	84.4	83.8	86.2	85	80.2	85.2	82.4	81.8	66.8	59.2	97.7 (3.0)
Fluency (10)	9	8	8	9	9	9	8	9	8	8	8	8	10.0 (0)
Information content (10)	10	8	7	8	8	8	6	9	7	8	5	4	9.7 (0.6)
Auditory comprehension (10)	10	9.7	8.4	8	9.7	8.2	9.7	8.6	9.3	9.4	7.6	6.9	9.8 (0.1)
Repetition (10)	8.4	10	10	8.2	8	9.7	8.4	8.8	9	8.4	9	7	9.9 (0.3)
Naming (10)	8.5	8.7	8.8	8.7	8.4	7.6	8	7.2	7.9	7.1	3.8	3.7	9.5 (0.6)
Reading (10)	9.8	9.8	9.8	8.9	9.7	9.7	8.5	6.8	9.8	8	7.3	4.6	9.5 (0.8)
Writing (10)	10	9.9	10	9.2	9.1	7	9.3	9.5	9.6	8.7	7	4.9	9.6 (1.0)
Praxis (60)	57	60	59	59	60	58	60	60	59	56	58	60	59.8 (0.7)
Calculation (24)	24	24	24	24	24	24	24	24	24	24	18	24	23.1 (2.3)
*Token test* (166)	166	160	155	155	155	154	165	159	159	144	142	111	163.6 (2.0)
*TLPA*													
Naming (200)	170	168	152	144	142	140	136	131	126	72	49	44	193.4 (5.4)
Comprehension (200)	194	200	194	193	200	198	191	197	179	161	175	98	199.4 (1.0)
Severity of word comprehension impairment	*	–	*	*	–	–	*	*	**	**	**	***	

The maximum score is noted in each row header. Severity of word comprehension impairment: –, normal; *, minimal; **, mild; ***, severe. SD, standard deviation; TLPA, Test of Lexical Processing in Aphasia; WAB, Western Aphasia Battery.

**Table 3:** Summary of language features and review based on the current classification criteria (Gorno-Tempini *et al*.)^5^

	Subjects											
	1	2	3	4	5	6	7	8	9	10	11	12
*Non-fluent/agrammatic variant*												
*Core features (one of two must be present)*												
Apraxia of speech	−	−	−	−	−	−	−	−	−	−	−	−
Agrammatism	−	−	−	−	−	−	−	−	−	−	−	−
*Other features (two of three must be present)*												
Impaired complex sentence comprehension	−	−	+	+	+	+	−	−	−	+	+	+
Spared single-word comprehension	−	+	−	−	+	+	−	−	−	−	−	−
Spared object knowledge	+	+	+	+	+	+	+	+	+	+	+	+
*Meets nfvPPA criteria?*	−	−	−	−	−	−	−	−	−	−	−	−
*Semantic variant*												
* Core features (both must be present)*												
Impaired confrontation naming	+	+	+	+	+	+	+	+	+	+	+	+
Impaired single-word comprehension	+	−	+	+	−	−	+	+	+	+	+	+
*Other features (three of four must be present)*												
Impaired object knowledge (e.g. identification of famous or familiar face, environmental sound recognition, and object use)	−	−	−	−	−	−	−	−	−	−	−	−
Surface dyslexia/dysgraphia	−	−	−	−	−	−	−	−	−	−	−	−
Spared repetition	−	+	+	−	−	+	−	−	−	−	−	−
Spared speech production	+	+	+	+	+	+	+	+	+	+	+	+
Meets svPPA criteria?	−	−	−	−	−	−	−	−	−	−	−	−
Logopenic variant												
*Core features (all must be present)*												
Impaired word retrieval	+	+	+	+	+	+	+	+	+	+	+	+
Impaired phrase repetition (e.g. <15 morae)	−	−	−	−	−	−	−	−	−	−	−	+
Impaired sentence repetition (e.g. ≥15 morae)	+	−	−	+	+	−	+	+	+	+	+	+
*Other features (three of four must be present)*												
Phonological errors	−	−	−	−	−	−	−	−	−	−	−	−
Spared word comprehension and object knowledge	−	+	−	−	+	+	−	−	−	−	−	−
	*		*	*			*	*	**	**	**	***
Spared motor speech	+	+	+	+	+	+	+	+	+	+	+	+
Absence of agrammatism	+	+	+	+	+	+	+	+	+	+	+	+
Meets lvPPA criteria?	−	−	−	−	+?	−	−	−	−	−	−	−
Redefine of aphasic features	A	A	A	A	A	A	A	A	T	T	T	W

Severity of word comprehension impairment: *, minimal; **, mild; ***, severe. A, anomic aphasia; lvPPA, logopenic variant of primary progressive aphasia; nfvPPA, non-fluent/agrammatic variant of primary progressive aphasia; svPPA, semantic variant of primary progressive aphasia; T, transcortical sensory aphasia; W, Wernicke’s aphasia.

All patients had anomia on the TLPA-naming task. When the phonemic cues were provided for the words that they could not name (typically the first one-letter or two-letters of the words), all patients generally produced the correct words. Therefore, phonemic cues were effective, suggesting the preservation of semantic memory.^41^ In addition, the words that were not produced during the official language examinations were sometimes produced on other tasks or another day, which does not support a pattern of consistent semantic deficits that is a hallmark of svPPA.^42^ Moreover, when the correct word was provided for word-finding difficulty on the naming task, no patients questioned the meaning of words (e.g. ‘What is camel?’), which was a specific diagnostic clue of svPPA.^43^ Single-word comprehension ability was within the normal range on the TLPA-word comprehension task in three patients (Cases 2, 5 and 6). Therefore, the diagnosis of these patients (Cases 2, 5 and 6) was not consistent with svPPA.^5^ None of the patients showed surface dysgraphia on the WAB-writing task or surface dyslexia on the ACE-R-reading task, which was considered to be a surrogate indicator of semantic memory deficits of svPPA.^44–47^ In addition, of the remaining nine patients, all except for one patient (Case 3) showed a decline on the WAB-Repetition task. Therefore, the diagnosis of eight patients (Cases 1, 4 and 7–12) was not consistent with svPPA.^5^ Moreover, none of the patients, including the patient in Case 3, showed non-verbal semantic memory deficits such as impaired famous-face knowledge on the VPTA, impairment of environmental sound recognition, impairment of object recognition or object misuse in daily activities. Because semantic memory, except for single-word comprehension, was relatively well preserved in all cases,^41–48^ the clinical presentations of the 12 patients were not sufficiently consistent with the diagnosis of svPPA (Table 3).^5^

Repetition ability was within the normal range on the WAB-Repetition task in three patients (Cases 2, 3 and 6). Therefore, the diagnosis was not consistent with the lvPPA in these cases.^5^ None of the patients showed speech production impairment, agrammatism or phonological errors in spontaneous speech and naming on the WAB and TLPA-naming tasks. In addition, the remaining nine patients except for one patient (Case 5) showed single-word comprehension impairment on the TLPA-word comprehension task. Therefore, the diagnosis of these eight patients (Cases 1, 4 and 7–12) was not consistent with lvPPA.^5^ The diagnosis of the remaining patient (Case 5) may be consistent with lvPPA.^5^ However, 11 patients (Cases 1–11), including Case 5, could correctly repeat the compound and polysyllabic words or phrases formed by up to four words of 14 morae on the WAB-Repetition task (‘*Nihon Koko yakyu remmei*’ ‘Japan High School Baseball Federation’). In addition, verbal short-term memory, as evaluated by the digit span, was well preserved in all (Cases 1–11) but one patient (Case 12). The clinical features of 11 patients (Cases 1–11), including Case 5, maybe atypical as the lvPPA diagnosis because the severity of the repetition deficit was mild.^49^ Our study was cross-sectional; hence, all patients may progress to typical svPPA or lvPPA. However, in the current study, we diagnosed the clinical features of the 12 patients at a given point in time as an unclassified fluent variant of PPA other than svPPA and lvPPA (Table 3).

### Lesion distribution on MRI and SPECT

**Figure 1:**
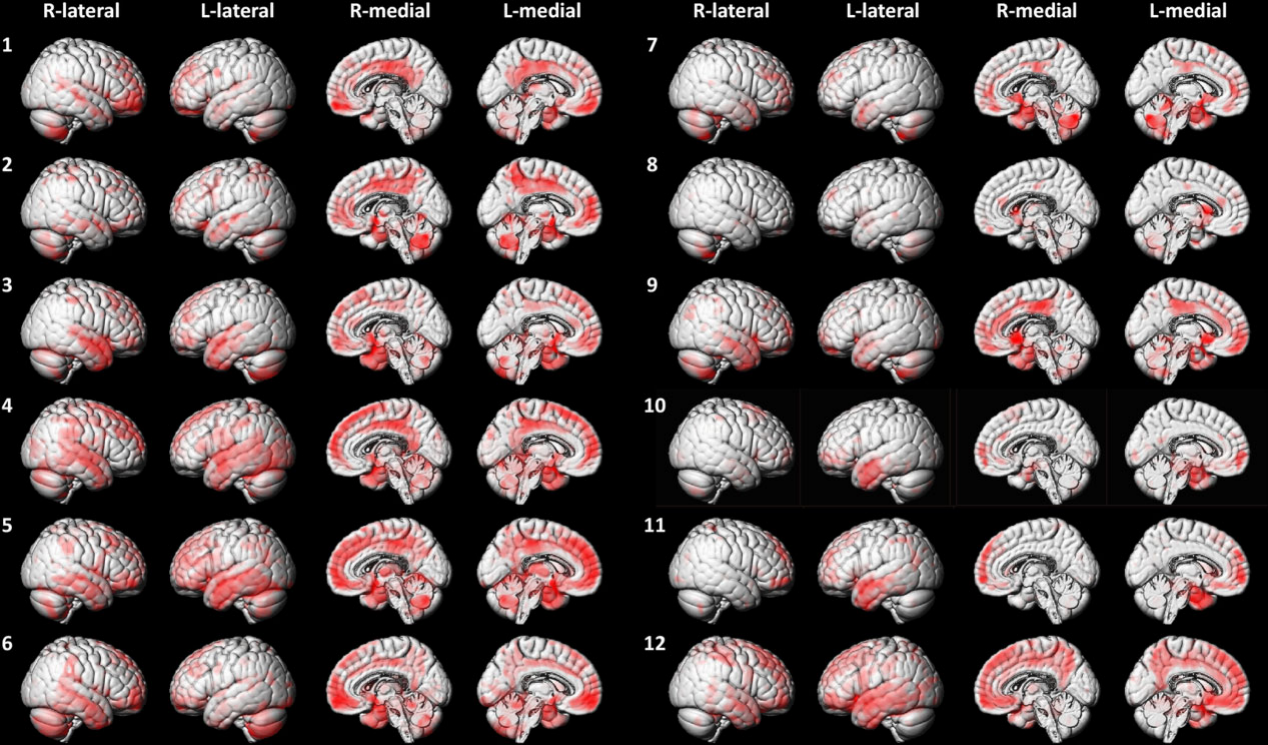
**Brain MRI**. Brain MRI analysed with the BAAD revealed patterns of significant atrophy in each of the 12 cases with an unclassified fluent variant of primary progressive aphasia. Case numbers are shown to the left of each set of images. Cases 1–8: progressive anomic aphasia; Cases 9–11: progressive TCSA; Case 12: progressive Wernicke’s aphasia. BAAD, Brain Anatomical Analysis using Diffeomorphic Anatomical Registration through Exponentiated Lie Algebra; L, left; R, right; TCSA, transcortical sensory aphasia.

Based on the BAAD analysis, MRI in the eight patients (Cases 1–8) revealed cortical atrophy accentuated in the temporoparietal lobes with no obvious lateralization (Fig. 1). MRI in one patient (Case 9) revealed atrophy, predominantly in the anterior cingulate and medial orbitofrontal cortex, with no obvious lateralization. MRI in three patients (Cases 10–12) revealed atrophy, predominantly in the left temporoparietal lobe. Atrophy in Case 12 was more extensive in the left frontal and temporoparietal lobes than in Cases 10 and 11.

**Figure 2:**
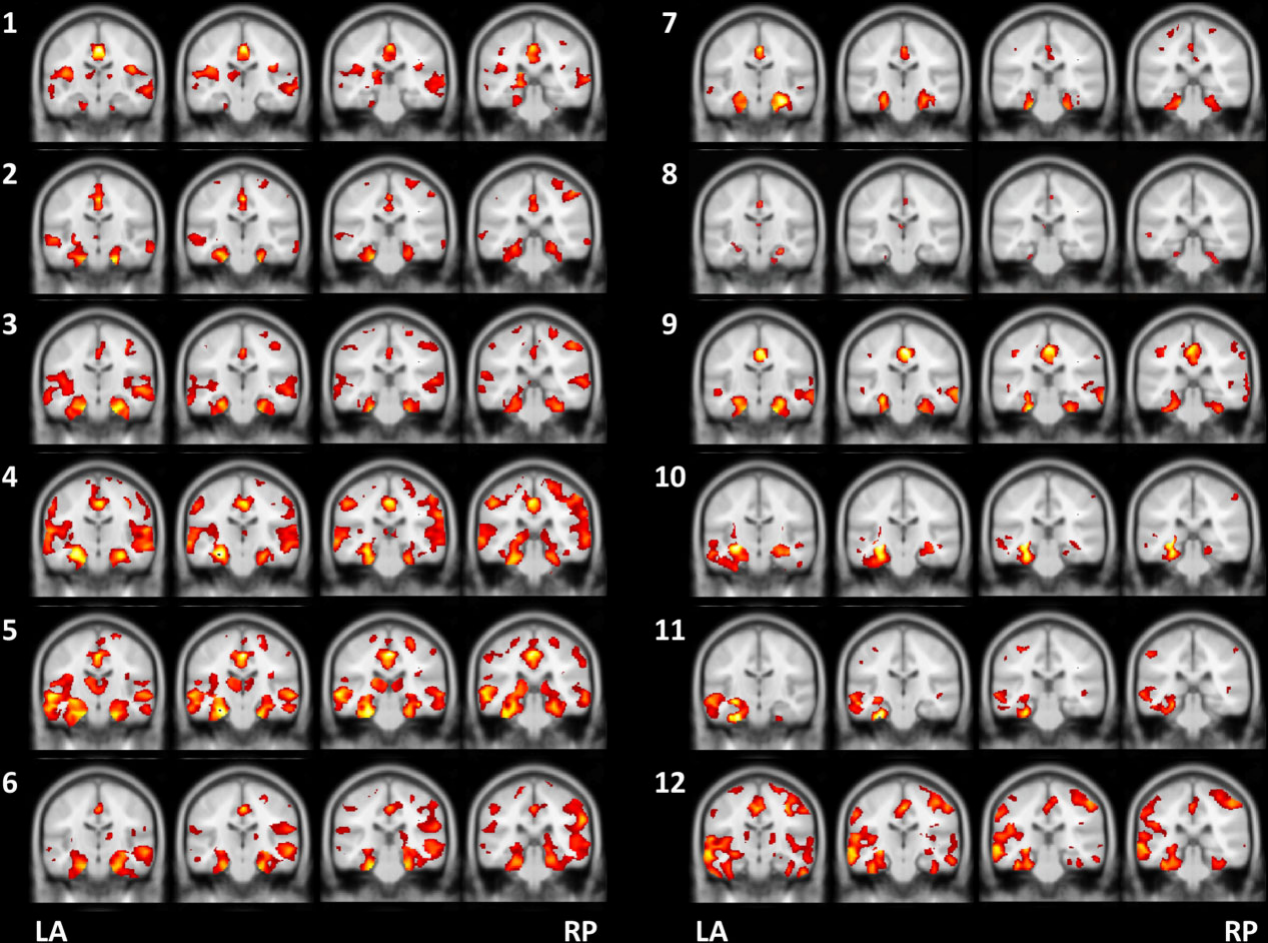
**Coronal brain MRI**. Analysis of coronal brain MR images with the BAAD revealed patterns of significant atrophy in each of the 12 cases with an unclassified fluent variant of primary progressive aphasia. Case numbers are shown on the left side of each set of images. Cases 1–8: progressive anomic aphasia; Cases 9–11: progressive TCSA; Case 12: progressive Wernicke’s aphasia. BAAD, Brain Anatomical Analysis using Diffeomorphic Anatomical Registration through Exponentiated Lie Algebra; LA, left anterior; RP, right posterior; TCSA, transcortical sensory aphasia.

Based on the ROI analysis on the BAAD, MRI in all patients revealed significant atrophy in the medial temporal lobe (Fig. 2; Supplementary Table 2); in the left, right, anterior or posterior part of the hippocampus in 11 patients (except for Case 1); and the left or both parts of the parahippocampus in all patients. In addition, atrophy in the posterior cingulate cortex was apparent in four patients (Cases 1, 4, 5 and 12), and in the precuneus in six patients (Cases 2–6 and 12).

**Figure 3:**
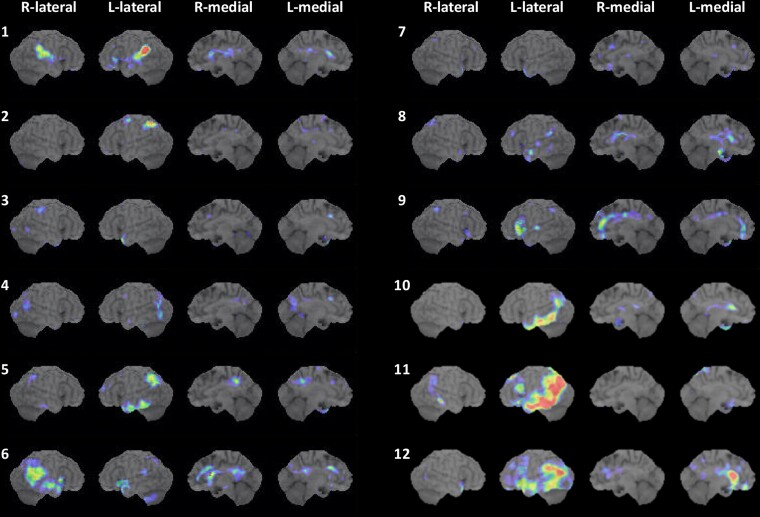
**Brain SPECT**. Brain SPECT analysed with 3D stereotactic surface projections revealed patterns of significant hypoperfusion in each of the 12 cases with an unclassified fluent variant of primary progressive aphasia. Case numbers are shown to the left of each set of images. Cases 1–8: progressive anomic aphasia; Cases 9–11: progressive TCSA; Case 12: progressive Wernicke’s aphasia. SPECT, single-photon emission computed tomography; L, left; R, right; TCSA, transcortical sensory aphasia.

Based on the 3D SSP analysis, SPECT revealed an area of hypoperfusion in the left hemisphere in the right-handed patients, and in the right hemisphere in the left-handed patient (Fig. 3). In addition, hypoperfusion was always noted in the bilateral or left unilateral posterior cingulate cortices and precuneus in nine patients (Cases 1, 2, 4–6 and 9–12). Hypoperfusion in the posterior cingulate cortex or precuneus was lacking in three patients (Cases 3, 7 and 8). The temporal lobe and the parietal lobe in the left hemisphere were involved in all patients but one; hypoperfusion in the right temporoparietal lobe was present in the left-handed patient (Case 6). Hypoperfusion was present in the left inferior frontal lobe in one patient (Case 9). In three patients (Cases 10–12), there was extensive hypoperfusion in the left frontal and temporoparietal lobes.

### Follow-up data of three patients

Three patients (Cases 6, 9 and 11) underwent follow-up examination after 1 year (Table 4). During Visit 2, spontaneous speech was found to be fluent in all three patients; there was no evidence of dysprosody, apraxia of speech or agrammatism. In Cases 6 and 9, repetition ability was within the normal range on the WAB-Repetition task, but the severity of the word comprehension impairment progressed to mild (Case 6) or severe (Case 9). Case 11 progressed to fluent aphasia with severe impairment of word comprehension and repetition. Case 11 also showed extensive deficits in cognitive domains other than language.

**Table 4:** Follow-up data of three patients

	Case 6		Case 9		Case 11		Normative data, mean (SD)
	Visit 1	Visit 2	Visit 1	Visit 2	Visit 1	Visit 2	
*Demographic characteristics*							
CDR total score (0–3)	0.5	0.5	0.5	0.5	0.5	1	
Duration from onset (years)	4	5	5	6	4	5	
Age at onset (years)	77		83		61		
MMSE total score (30)	19	17	16	17	14	4	28.6 (1.4)
*Types of aphasia*	A	T	T	T	T	W	
*WAB*							
Aphasia quotient (100)	85	81.6	82.4	84	66.8	52.8	97.7 (3.0)
Fluency (10)	9	8	8	8	8	8	10.0 (0)
Information content (10)	8	8	7	8	5	5	9.7 (0.6)
Auditory comprehension (10)	8.2	7.8	9.3	8.6	7.6	5.8	9.8 (0.1)
Repetition (10)	9.7	9.6	9	9.6	9	6.4	9.9 (0.3)
Naming (10)	7.6	7.4	7.9	7.8	3.8	1.2	9.5 (0.6)
Reading (10)	9.7	7.9	9.8	9	7.3	4.8	9.5 (0.8)
Writing (10)	7	7.8	9.6	7.9	7	3.1	9.6 (1.0)
Praxis (60)	58	60	59	59	58	41	59.8 (0.7)
Calculation (24)	24	24	24	24	18	3	23.1 (2.3)
*Token test* (166)	154	126	159	150	142	24	163.6 (2.0)
*TLPA*							
Naming (200)	140	120	126	126	49	17	193.4 (5.4)
Comprehension (200)	198	174	179	157	175	132	199.4 (1.0)
		**	**	***	**	***	

The maximum score is noted in each row header. Severity of word comprehension impairment: *, minimal; **, mild; ***, severe. A, anomic aphasia; CDR, Clinical Dementia Rating; MMSE, Mini-Mental State Examination; SD, standard deviation; T, transcortical sensory aphasia; TLPA, Test of Lexical Processing in Aphasia; WAB, Western Aphasia Battery; W, Wernicke’s aphasia.

## Discussion

The present study demonstrated that the clinical and imaging features of the 12 patients met the core criteria for PPA, but did not meet the current criteria for classification into any variants of PPA.^5^ All patients were diagnosed with an unclassified fluent variant of PPA. Based on the classical concept of progressive fluent aphasia, we redefined the diagnoses of 12 patients with an unclassified PPA as the following: primary progressive anomic aphasia (eight patients: Cases 1–8), primary progressive TCSA (three patients: Cases 9–11) and primary progressive Wernicke’s aphasia (one patient: Case 12) explained in detail below. These findings, suggesting the existence of unclassified fluent variants of PPA that are distinct from svPPA or lvPPA, provide further insights into the spectrum of PPA.

In our study, language functions were predominantly affected in the 12 patients, but other cognitive functions, such as memory and visuoperceptual and visuospatial function, remained relatively well preserved. The 12 patients showed no apraxia of speech or agrammatism which are characteristic symptoms of nfvPPA, no non-verbal semantic memory deficits or surface dyslexia/dysgraphia which were characteristic symptoms of svPPA, and no phonological errors which were regarded as a characteristic symptom of lvPPA. Under the current criteria for the classification of PPA, anomia (impaired confrontation naming in svPPA/impaired word retrieval in lvPPA) is one of the two core features of svPPA and lvPPA. However, anomia was observed in all aphasic patients. Therefore, in cases of no characteristic symptoms for any PPA variants, the diagnosis of svPPA and lvPPA relies on the presence of the other core feature (single-word comprehension impairment for svPPA/sentence repetition impairment for lvPPA), because anomia is not a specific symptom for svPPA and lvPPA. In two patients (Cases 2 and 6) with both TLPA-word comprehension and WAB-Repetition scores within the normal range, the deficits were fully compatible with anomic aphasia.^13,14,16,19,20^ In two patients (Cases 3 and 5) with either lower TLPA-word comprehension or WAB-Repetition scores compared to healthy individuals, the clinical symptoms may meet the core features of svPPA (for Case 3) or lvPPA (for Case 5). However, the deficit severity was negligible. In particular, mild impairment of sentence repetition as the core feature of lvPPA is also often caused by attentional deficits observed in other neurological disorders and therefore may not be a sufficiently specific symptom for lvPPA. Regarding clinical judgements of the PPA caused by neurodegenerative diseases, considering not only the presence or absence of symptoms (all or none) but also the severity of symptoms (more or less) may lead to a better understanding of the PPA syndromes. These subtle deficits consistent with different degrees of atrophy of the left hemisphere are a common finding observed in multiple dementia syndromes including non-aphasic Alzheimer’s disease.^50^ Moreover, several recent studies have demonstrated mild impairments of semantic processing in a proportion of cases of lvPPA^50^ as well as mild impairments of sentence repetition in a proportion of cases of svPPA,^49^ which are supported by the magnitude of atrophy. In the remaining eight patients (Cases 1, 4 and 7–12) with lower TLPA-word comprehension and WAB-Repetition scores than healthy individuals, although the clinical presentation did not meet the current criteria for typical svPPA and lvPPA,^5^ the clinical presentation may reflect the heterogeneity of svPPA and lvPPA. In addition, our study was cross-sectional; hence, anomic aphasia or unclassified fluent variants of PPA may progress to typical svPPA or lvPPA.^12,51^ However, the features of anomic aphasia and unclassified fluent variants of PPA at a given time point in the 12 patients were distinct from those of typical svPPA and lvPPA.

**Figure 4:**
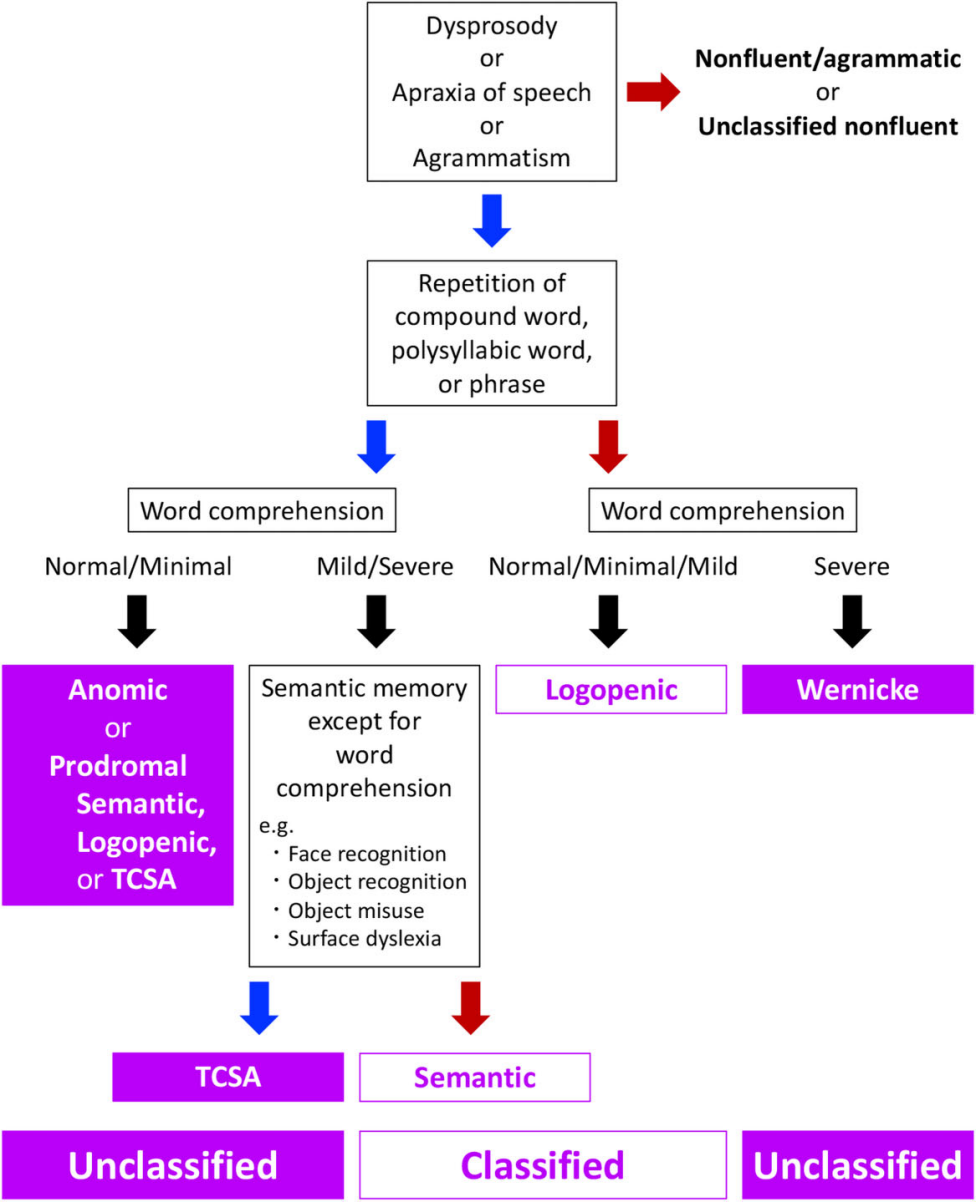
**Operational classification criteria**. The TLPA-auditory word comprehension test was used to classify the severity of word comprehension impairment. Normal/minimal: TLPA-auditory word comprehension scores 181–200; mild: 161–180; severe: <161. Red arrow indicates ‘significant’. Blue arrow indicates ‘insignificant’. ‘Significant’ and ‘insignificant’ impairments were qualitatively assessed based on the clinical examinations or daily activities. TCSA, transcortical sensory aphasia; TLPA, Test of Lexical Processing in Aphasia.

Before the research criteria of PPA^5^ were proposed, previous studies showed that the feature of language impairment in Alzheimer’s disease was progressive anomic aphasia in the early stage and progressive TCSA or Wernicke’s aphasia in the late stage.^10,11^ Both anomic aphasia and TCSA are fluent aphasias characterized by relatively preserved repetition. According to the WAB, the WAB-Repetition score of anomic aphasia/TCSA was ≥8.^52^ In our study, the language deficit may be classified as anomic aphasia/TCSA in 11 patients (Cases 1–11) and Wernicke’s aphasia in one patient (Case 12). Due to impaired word comprehension in addition to naming deficits, TCSA may be considered a more appropriate classification for three patients (Cases 9–11). TCSA is also similar to svPPA or aphasia observed in semantic dementia because these aphasic syndromes are characterized by fluent speech, relatively preserved repetition and impaired word comprehension;^41,53^ indeed, the classical concept ‘*Gogi* (word meaning) aphasia’ observed in semantic dementia^54^ has been historically placed in the category of TCSA.^55^ Because both svPPA and semantic dementia are caused by semantic memory deficits, patients generally indicate not only impaired single-word comprehension but also other multimodal deficits that reflect a loss of semantic knowledge, such as surface dyslexia/dysgraphia, impairment of environmental sound recognition, visual impairments in face and object recognition, or object misuse.^1,44–48,56–60^ However, the three patients (Cases 9–11) did not show symptoms that reflect the loss of semantic memory except for single-word comprehension. Moreover, both effectiveness of phonemic cues on the naming tasks^41^ and inconsistency of the semantic deficits for the same words^42^ may also reflect the preservation of semantic memory. In addition, no patients questioned the meaning of words (e.g. ‘What is camel?’), which was a specific diagnostic clue for svPPA and semantic dementia, when the correct word was provided for word-finding difficulty on the naming task.^43^ Thus, in terms of the preservation of semantic memory, the three patients (Cases 9–11) were distinguished from svPPA and semantic dementia. Therefore, based on the classical findings of progressive fluent aphasias in Alzheimer’s disease,^8–11^ we may be able to re-categorize the diagnoses as follows: primary progressive anomic aphasia, eight patients (Cases 1–8); primary progressive TCSA, three patients (Cases 9–11); and primary progressive Wernicke’s aphasia, one patient (Case 12) (Fig. 4). However, we did not directly compare the features of aphasia caused by stroke with those caused by neurodegeneration. Ingram *et al*.^22^ compared the features of aphasia caused by stroke with those caused by neurodegeneration and found that most patients with svPPA or lvPPA were more fluent than patients with anomic aphasia caused by stroke. Although we followed the terminology of the classification of aphasia following stroke in the current study based on the classical findings of progressive fluent aphasias in Alzheimer’s disease,^8–11^ further investigations are warranted to confirm the differences between anomic aphasia/TCSA/Wernicke’s aphasia caused by stroke and those caused by neurodegeneration.

The lesions most relevant to the language impairment, noted as cortical atrophy on MRI and as hypoperfusion on SPECT, were located in the temporal and/or parietal cortices in the dominant hemisphere in most cases. In primary progressive anomic aphasia (Cases 1–8), unlike svPPA,^5^ the affected area varies across cases but is never limited to the anterior temporal lobe. In primary progressive TCSA (Cases 9–11), the affected area included the inferior frontal cortex in one patient (Case 9) and the temporoparietal cortices, with Wernicke’s area seemingly spared in two patients (Cases 10 and 11). In primary progressive Wernicke’s aphasia (Case 12), the temporoparietal cortices, including Wernicke’s area, were involved. These regions are known to cause the corresponding types of aphasia. The involvement of the left inferior frontal lobe in one patient with TCSA (Case 9) represents an interesting finding. Stroke lesions in this region can cause TCSA,^53^ which may also be caused by neurodegeneration.

Regarding the aetiologies of PPA, the underlying causes of unclassified fluent variants of PPA remain unclear. Although progressive fluent aphasias other than svPPA and lvPPA are observed in patients with Alzheimer’s disease,^8–11,61,62^ none of our patients met the National Institute on Aging and Alzheimer’s Association diagnostic criteria for Alzheimer’s disease,^63^ which includes a progressive decline in two or more cognitive domains, including memory, as mandatory items. Although lvPPA has been proposed as an atypical Alzheimer’s disease in the International Working Group-2 research criteria for Alzheimer’s disease,^64^ fluent variants of PPA other than lvPPA have not been included. Nevertheless, neuroimaging supported the hypothesis that Alzheimer’s disease is a likely underlying disease in most cases. The BAAD analysis on MRI revealed significant atrophy in the parahippocampus, including the entorhinal cortex, in 12 patients, and in the hippocampus in 11 patients, which is characteristic of the early stages of Alzheimer’s disease.^65^ In addition, the 3D SSP analysis on SPECT revealed significantly reduced blood flow in the posterior cingulate gyrus and/or precuneus in nine patients, which also reflects one of the surrogate markers of early Alzheimer’s disease.^66^ Moreover, amyloid imaging was performed in only one patient (Case 11), with positive results. However, two patients (Cases 7 and 8) had neither atrophy nor hypoperfusion in the posterior cingulate gyrus and precuneus. In previous studies of progressive aphasia, a proportion of patients with progressive fluent aphasia with a predominant anomic feature,^13,19,20^ was amyloid PET-negative.^19^ Therefore, neurodegenerative diseases other than Alzheimer’s disease, such as corticobasal degeneration,^67^ Lewy body disease,^68,69^ or transactive response DNA binding protein 43 accumulation,^70,71^ should be considered in some cases.

Language disturbances in Alzheimer’s disease begin with word-finding difficulty, which progresses to anomic aphasia.^11,72,73^ Thereafter, anomic aphasia progresses to TCSA with impaired comprehension, but retained repetition (consistent with Case 6).^9–11^ Then, TCSA advances to Wernicke’s aphasia with impaired comprehension and repetition (consistent with Case 11).^11^ This is the reverse of the recovery from Wernicke’s aphasia, via TCSA, to anomic aphasia in aphasics after stroke.^74^ Other patterns of progression are from anomic aphasia to lvPPA with impaired repetition^12^ and from lvPPA to Wernicke’s aphasia with impaired comprehension.^75–77^ This is the reverse of another recovery pattern from Wernicke’s aphasia to conduction aphasia or to anomic aphasia after stroke.^78–80^ However, this pattern of progression could be observed only in limited cases of primary progressive TCSA or Wernicke’s aphasia, where the deficit remains in the language domain. In the majority of the cases, the disease is expressed as primary progressive anomic aphasia, and as the disease progresses, extensive aphasia is no longer recognized as primary progressive TCSA or Wernicke’s aphasia due to the emerged generalized cognitive deficits (consistent with Case 11). This is likely to be the reason why primary progressive Wernicke’s aphasia, primary progressive TCSA and primary progressive anomic aphasia are less prevalent, in that order.

The present study has several limitations. First, the design of our study was cross-sectional, and only three patients could be followed up. As mentioned above, we postulated that there was an association between primary progressive anomic aphasia and the development of other PPAs (svPPA, lvPPA, progressive TCSA and progressive Wernicke’s aphasia); however, the association was not clear in the present study. This hypothesis must be corroborated in a prospectively followed cohort. In addition, the rate of disease progression and the time to the emergence of additional deficits should be confirmed for providing staged counselling and care support. Second, our patients did not undergo additional intensive tests (except for the TLPA) of oral-motor, phonological and semantic function such as loss of object knowledge and surface dyslexia or dysgraphia, although we confirmed that none of the patients had severe impairments, based on comprehensive qualitative tests by an experienced behavioural neurologist and speech and language therapist. It cannot be overlooked that the initial presentation or forme fruste of the typical variants of PPA (nfvPPA, svPPA, and lvPPA) may have looked like anomic aphasia/TCSA because pathognomonic deficits were too subtle to be detected. Furthermore, specific issues in Japanese aphasic patients such as differences in the spectrum of syllabification affecting repetitions or reading and writing systems [kanji (morphograms)/kana (phonograms)] should be addressed in future studies. Moreover, the WAB, which was developed for assessing aphasia following stroke, has been widely used in assessing language deficits caused by neurodegenerative diseases. However, some studies have demonstrated that the WAB alone is insufficient to detect the characteristic symptoms to discriminate among PPA variants.^20,33^ The development of standardized quantitative measures for distinguishing multiple variants of PPA should be incorporated in future studies. Third, CSF and PET biomarkers were not examined or were examined in only one case. Moreover, neuropathological examinations were not available. Therefore, further investigations are needed to determine the aetiologies of aphasia.

In conclusion, the present study demonstrated the clinical and imaging features of 12 patients with an unclassified fluent variant of PPA. Before the research criteria of PPA^5^ were proposed, progressive anomic aphasia in the early stage and progressive TCSA or Wernicke’s aphasia in the late stage were frequently reported as the feature of language impairment in Alzheimer’s disease.^8–11^ Based on these concepts, we reconsider the existence of classical primary progressive fluent aphasias (which we defined as primary progressive anomic aphasia, primary progressive TCSA and primary progressive Wernicke’s aphasia), which are distinct from svPPA or lvPPA, and provide insights into the spectrum of PPA.

## Supplementary Material

fcac015_Supplementary_Data

## Abbreviations

ACE-R =: Addenbrooke’s Cognitive Examination-Revised
ADAS =: Alzheimer’s Disease Assessment Scale
BAAD =: Brain Anatomical Analysis using Diffeomorphic Anatomical Registration through Exponentiated Lie Algebra
CDR =: Clinical Dementia Rating
DARTEL =: Diffeomorphic Anatomical Registration through Exponentiated Lie Algebra
FTD =: frontotemporal dementia
lvPPA =: logopenic variant of primary progressive aphasia
MNI =: Montreal Neurological Institute
nfvPPA =: non-fluent/agrammatic variant of primary progressive aphasia
NPI =: Neuropsychiatric Inventory
PPA =: primary progressive aphasia
ROI =: region of interest
SD =: standard deviation
SPECT =: single-photon emission computed tomography
SPM =: statistical parametric mapping
SSP =: stereotactic surface projection
svPPA =: semantic variant of primary progressive aphasia
TCSA =: transcortical sensory aphasia
TLPA =: Test of Lexical Processing in Aphasia
VBM =: voxel-based morphometry
VPTA =: Visual Perception Test for Agnosia
WAB =: Western Aphasia Battery
