# Supplementary material for: Unclassified fluent variants of primary progressive aphasia: distinction from semantic and logopenic variants
Source: Brain Commun. 2022 Feb 2;4(3):fcac015. doi: 10.1093/braincomms/fcac015 (PMC9171501; doi:10.1093/braincomms/fcac015)
Supplement: fcac015_Supplementary_Data [file fcac015_supplementary_data.docx]

**Supplementary material**

**Supplementary Table 1. Performance in neuropsychological and behavioural tests**

|  | Case No. | | | | | | | | | | | | Normative data, mean (SD) |
| --- | --- | --- | --- | --- | --- | --- | --- | --- | --- | --- | --- | --- | --- |
|  | 1 | 2 | 3 | 4 | 5 | 6 | 7 | 8 | 9 | 10 | 11 | 12 |  |
| **ACE-R** |  |  |  |  |  |  |  |  |  |  |  |  |  |
| Total [100] | 87 | 78 | **73** | **63** | **61** | **62** | **65** | **47** | **63** | **49** | **44** | **38** | 91.1 (8.4) |
| Attention/Orientation [18] | 18 | 17 | 17 | **15** | **13** | **10** | **12** | **10** | **15** | **10** | **9** | **6** | 17.5 (1.2) |
| Memory [26] | 22 | 17 | **11** | **6** | **6** | **7** | **10** | **3** | **8** | **6** | **5** | **4** | 21.0 (4.8) |
| Verbal fluency [14] | 9 | **4** | 10 | **7** | 10 | **4** | 9 | **3** | **8** | **4** | **3** | **2** | 12.3 (2.0) |
| Language [26] | 22 | 24 | **19** | **19** | **16** | 25 | **18** | **15** | **16** | **13** | **11** | **10** | 24.3 (1.7) |
| Visuospatial [16] | 16 | 16 | 16 | 16 | 16 | 16 | 16 | 16 | 16 | 16 | 16 | 16 | 15.9 (0.2) |
| **ADAS** |  |  |  |  |  |  |  |  |  |  |  |  |  |
| Total [0–70] | 5.1 | 8.7 | 6.4 | 8.1 | **11.4** | **18.1** | 9.8 | **19.1** | **12.4** | **15.7** | **27** | **32.7** | 5.5 (2.6) |
| Recall [30] | 22 | 16 | **13** | **11** | **11** | **7** | **14** | **13** | 18 | **8** | **9** | **8** | 21.5 (2.8) |
| Recognition [36] | 29 | 30 | 34 | 34 | 33 | 29 | 35 | 26 | 29 | 32 | 28 | 29 | 31.3 (3.9) |
| **VPTA famous face** [0-24] | 0 | 0 | 0 | 0 | 0 | 0 | 0 | 0 | 0 | 0 | 0 | 0 | 0.04 (0.1) |
| **NPI** |  |  |  |  |  |  |  |  |  |  |  |  |  |
| Persecutory delusions | 0 | 0 | 0 | 0 | 0 | 0 | 0 | 0 | 0 | 0 | 0 | 0 |  |
| Delusional misidentifications | 0 | 0 | 0 | 0 | 0 | 0 | 0 | 0 | 0 | 0 | 0 | 0 |  |
| Hallucinations | 0 | 0 | 0 | 0 | 0 | 0 | 0 | 0 | 0 | 0 | 0 | 0 |  |
| Agitation/aggression | 0 | 0 | 0 | 0 | 0 | 0 | 0 | 0 | 0 | 0 | 0 | 0 |  |
| Depression | 0 | **1** | 0 | 0 | 0 | 0 | 0 | 0 | 0 | 0 | 0 | 0 |  |
| Anxiety | 0 | 0 | 0 | 0 | 0 | 0 | 0 | 0 | 0 | **1** | 0 | 0 |  |
| Euphoria | 0 | 0 | 0 | 0 | 0 | **4** | 0 | 0 | 0 | 0 | 0 | 0 |  |
| Apathy | 0 | 0 | 0 | 0 | 0 | 0 | 0 | 0 | 0 | **8** | 0 | 0 |  |
| Disinhibition | 0 | 0 | 0 | 0 | 0 | 0 | 0 | 0 | 0 | 0 | 0 | 0 |  |
| Irritability/lability | 0 | 0 | 0 | 0 | 0 | **1** | 0 | 0 | 0 | 0 | 0 | 0 |  |
| Aberrant motor behaviour | 0 | 0 | 0 | 0 | 0 | 0 | 0 | 0 | 0 | 0 | 0 | 0 |  |
| Sleep disturbances | 0 | 0 | 0 | 0 | 0 | 0 | 0 | 0 | 0 | 0 | 0 | 0 |  |
| Eating abnormalities | 0 | 0 | 0 | 0 | 0 | 0 | 0 | 0 | 0 | 0 | 0 | 0 |  |
| Fluctuations in cognition | 0 | 0 | 0 | 0 | 0 | 0 | 0 | 0 | 0 | 0 | 0 | 0 |  |

**Abbreviations:** ACE-R = Addenbrooke’s Cognitive Examination-Revised; NPI = Neuropsychiatric Inventory; SD = standard deviation; VPTA = Visual Perception Test for Agnosia.

The maximum score is noted in each row header.

Boldfacing represents values that are considered abnormal.

**Supplementary Table 2. Z values in each region of interest**

|  | Subjects | | | | | | | | | | | |
| --- | --- | --- | --- | --- | --- | --- | --- | --- | --- | --- | --- | --- |
| ROI | 1 | 2 | 3 | 4 | 5 | 6 | 7 | 8 | 9 | 10 | 11 | 12 |
| Hippocampus |  |  |  |  |  |  |  |  |  |  |  |  |
| Left anterior | 1.569 | **2.804** | **3.793** | **5.223** | **4.362** | **4.216** | **3.399** | **2.038** | **3.959** | **3.689** | **3.278** | **2.752** |
| Left posterior | 1.509 | 1.427 | 1.17 | **3.359** | **3.63** | **2.266** | 1.326 | 1.234 | 1.367 | **4.089** | **2.106** | **2.669** |
| Right anterior | 0.036 | **2.111** | **3.263** | **3.853** | **3.392** | **5.341** | **4.414** | **2.034** | **4.125** | **3.452** | 1.403 | **2.346** |
| Right posterior | -0.986 | 0.82 | 0.2 | 1.269 | **2.465** | **2.59** | 1.098 | -0.074 | 0.204 | 1.283 | 1.15 | 0.947 |
| Parahippocampus |  |  |  |  |  |  |  |  |  |  |  |  |
| Left | **2.87** | **4.149** | **5.172** | **5.349** | **5.497** | **5.33** | **4.154** | **2.058** | **4.667** | **3.912** | **4.36** | **3.121** |
| Right | 0.427 | **3.281** | **4.489** | **4.267** | **4.547** | **4.899** | **3.878** | 1.128 | **4.087** | **2.12** | 1.608 | **2.227** |
| Posterior cingulum |  |  |  |  |  |  |  |  |  |  |  |  |
| Left | **3.448** | 1.819 | 0.008 | **2.595** | **2.959** | 1.367 | 0.809 | -1.21 | 1.6 | 1.911 | 0.275 | **2.814** |
| Right | 1.996 | 0.388 | -0.044 | **2.547** | 1.339 | 1.14 | 0.568 | -2.297 | 0.911 | -0.207 | -0.745 | 1.337 |
| Precuneus |  |  |  |  |  |  |  |  |  |  |  |  |
| Left | -0.184 | **3.071** | **2.421** | **2.543** | **3.529** | **2.956** | 1.366 | -3.245 | 0.801 | 0.412 | 1.619 | **4.427** |
| Right | 0.212 | **2.014** | 1.635 | **2.39** | **3.072** | **2.882** | 0.804 | -1.246 | 0.64 | -0.605 | 0.686 | **3.255** |

**Abbreviation:** ROI = region of interest; Boldfacing represents abnormal values in reference to normative data (2 standard deviations below the mean).
